# Who Owns the Data? Open Data for Healthcare

**DOI:** 10.3389/fpubh.2016.00007

**Published:** 2016-02-17

**Authors:** Patty Kostkova, Helen Brewer, Simon de Lusignan, Edward Fottrell, Ben Goldacre, Graham Hart, Phil Koczan, Peter Knight, Corinne Marsolier, Rachel A. McKendry, Emma Ross, Angela Sasse, Ralph Sullivan, Sarah Chaytor, Olivia Stevenson, Raquel Velho, John Tooke

**Affiliations:** ^1^Department of Computer Science, University College London (UCL), London, UK; ^2^Parliamentary Office of Science and Technology, London, UK; ^3^Department of Clinical and Experimental Medicine, University of Surrey, Guildford, and Royal College of General Practitioners Research and Surveillance Centre, London, UK; ^4^Faculty of Population Health Sciences, University College London (UCL), London, UK; ^5^London School of Hygiene & Tropical Medicine, London, UK; ^6^University College London Partners (UCLP), London, UK; ^7^Department of Health, London, UK; ^8^Cisco Consulting Services, Life Sciences, Health and Care, Paris, France; ^9^The London Centre for Nanotechnology and Division of Medicine, University College London (UCL), London, UK; ^10^Chatham House Centre on Global Health Security, London, UK; ^11^Health Informatics Group, Royal College of General Practitioners, London, UK; ^12^UCL Public Policy, UCL, London, UK; ^13^Department of Science and Technology Studies, UCL, London, UK; ^14^School of Life and Medical Sciences, UCL, London, UK

**Keywords:** open data, data ownership, healthcare EPR, privacy, data policy

## Abstract

Research on large shared medical datasets and data-driven research are gaining fast momentum and provide major opportunities for improving health systems as well as individual care. Such open data can shed light on the causes of disease and effects of treatment, including adverse reactions side-effects of treatments, while also facilitating analyses tailored to an individual’s characteristics, known as personalized or “stratified medicine.” Developments, such as crowdsourcing, participatory surveillance, and individuals pledging to become “data donors” and the “quantified self” movement (where citizens share data through mobile device-connected technologies), have great potential to contribute to our knowledge of disease, improving diagnostics, and delivery of ­healthcare and treatment. There is not only a great potential but also major concerns over privacy, confidentiality, and control of data about individuals once it is shared. Issues, such as user trust, data privacy, transparency over the control of data ownership, and the implications of data analytics for personal privacy with potentially intrusive inferences, are becoming increasingly scrutinized at national and international levels. This can be seen in the recent backlash over the proposed implementation of care.data, which enables individuals’ NHS data to be linked, retained, and shared for other uses, such as research and, more controversially, with businesses for commercial exploitation. By way of contrast, through increasing popularity of social media, GPS-enabled mobile apps and tracking/wearable devices, the IT industry and MedTech giants are pursuing new projects without clear public and policy discussion about ownership and responsibility for user-generated data. In the absence of transparent regulation, this paper addresses the opportunities of Big Data in healthcare together with issues of responsibility and accountability. It also aims to pave the way for public policy to support a balanced agenda that safeguards personal information while enabling the use of data to improve public health.

## Introduction

The recent emergence of Big Data in healthcare [including large linked data from electronic patient records (EPR) as well as streams of real-time geo-located health data collected by personal wearable devices, etc.] and the open data (movement enabling sharing datasets) are creating new challenges around ownership of personal data while opening new research opportunities and drives for commercial exploitation (1). A balance must be struck between an individual’s desire for privacy and their desire for good evidence to drive healthcare, which may sometimes be in conflict. Opportunities for research on EPR and public health medical datasets have already demonstrated impressive results in generating new evidence (2); however, new computer science approaches analyzing real-time Big Data streams generated by social media and increasingly popular tracking/wearable devices have re-charted the data ownership landscape. And dramatically accelerated computing research activities into pilots demonstrating improving personal health outcomes and disease management through knowledge discovery and personalized medicine (3, 4) to provide signals for early warning for outbreaks and pandemics (5, 6) as well as to track citizens response (7).

With the increasing use of mobile and wearable devices (8), new opportunities were created for personalized health (tailored care to the needs of an individual) (9), crowdsourcing, participatory surveillance, and movement of individuals pledging to became “data donors” and the “quantified self” initiative^1^ (where citizens share data through mobile device-connected technologies). These initiatives created large volumes of data with considerable potential for research through open data initiatives. However, user privacy and ownership of user-generated data remain an under-explored territory from policy and regulatory perspectives while becoming a booming business for social media industry and MedTech manufactures (10).

Therefore, in the absence of transparent data ownership regulation, two strikingly disparate approaches emerged for data ownership, usage, and responsibility over sharing and accountability: first, government-regulated clinical and research medical data (including individual and population data gathered by non-government organizations in high- and low-income settings) and, second, private user-generated health data collected from social media, apps, online searches and wearable devices.

First, poor government communication, unclear agendas, and lack of transparency over the control and ownership of medical data are increasingly scrutinized at national and international levels. For example, in the UK, those hoping to deliver improved healthcare on the back of better access to individual data have failed to gain citizens’ trust. This was seen in the backlash over the proposed implementation of the care.data initiative in the UK (11) intended to enable large NHS individual data sharing with researchers and, controversially, with businesses which resulted in a failure of the initiative (12).

Second, some citizens seem little concerned over their much more accurate and potentially private user-generated health data being directly collected by IT and social media companies and MedTech manufactures through tracking/wearable devices and social media with commonly no opt-out options, potentially subject to personal intrusion using data analytics driven marketing and unregulated sharing and use (13).

However, this observation raises interesting questions: what are the motivations of citizens who are at the intersection of these two groups and what is the size of this “contradicting” population? Could this behavior be explained by simply a lack of awareness of seamless data collection (13)? Or is the mater more complex: there might be citizens feeling that their explicit consent is required for data sharing in the former group (where the data are *extracted* from clinical records to be shared), while they agreed to sharing with IT and MedTech industry in the second group (where the user-generated content could be considered *donated* by accepting terms and conditions). Better understanding of citizens’ motivations requires further research, especially as many terms of condition often provide no opt-out options. Nevertheless, perhaps more important than ownership and consent for sharing data, is the question of: *by whom* and for *what purposes* are shared citizens data *used* and how could decisions be effectively controlled by citizens themselves?

## Benefits of Opening up Health Data for Research

At the clinical/population and research data level, opening up medical data, sharing and linking large healthcare datasets enables semantically to relate and enrich data on symptoms, diseases, diagnosis, treatments, and prescriptions offering the potential for improvements in care for individuals and populations as well more efficient semantic access to the evidence base (14, 15). Linking datasets further enhances this potential, helping to produce new evidence, discovery of unknown symptoms and personal treatments, and better understanding of health outcomes and healthcare delivery challenges. This is invaluable for policymakers (e.g., geographical analysis of antibiotics prescription rates) as well as enabling more efficient ways of working for healthcare practitioners (e.g., automated repeated prescriptions have been reducing GP consultation time) (16). However, while many service providers and users are happy to see their data shared for reasons of altruism, there is recognition that there may also have to be more immediate benefits for individuals and practitioners, and clearer communication of those benefits (17).

From citizens’ perspective benefits come with, for example, better understanding of specific diseases, improvements in care for long-term conditions, and opportunities for home care using remote and telehealth technologies enabled by easier access to information. Though while record systems are being opened up to individuals, beyond specialist areas of care there has not been enormous uptake or clear demonstration of the benefits (18).

There are also benefits from sharing information on social inequities and population health at all levels: globally (for example, comparing low- and high-income countries), nationally, and locally (e.g., class inequity). The sharing of large population level data helps researchers to accurately describe these inequities and highlight problematic areas, specific target groups, and geographical and regional challenges to be addressed through new sets of evidence-based dedicated health interventions.

The Internet of Things (IoT) is part of the era of the “Internet of Everything” – computers, data, processes, sensors, people, wearable, and tracking medical devices (soon 50 billions of smart objects worldwide) are being connected to the Internet and use distributed cloud-based data storage infrastructures (19). These technological advances created an unprecedented level of personal data sharing from wearable medical devices, social media, and personal fitness tracking, to loyalty cards recording our shopping habits. New algorithms for Big Data mining and analytics investigating streams of real-time personalized time/geo-located data sources provide new opportunities for personalized health advice, monitoring, and treatment of specific conditions as well as increasing wellbeing (20).

Finally, there are other large datasets potentially benefiting from research: government population level epidemiological datasets collected through surveillance systems. There are successful moves in this direction, such as the UK national influenza surveillance program [Royal College of General Practitioners Research and Surveillance Centre (21)], however, the ideal case for research exploitation of population level data with no privacy concerns would be enabling access to datasets in machine readable format, championed by the Linked Data initiative (22). At international level, sharing even historical population level data remains a challenge. While disease risk notifications are legally defined by WHO at international level (International Health Regulations, IHR) and ECDC (EC Decision 2008/426/EC) countries remain in control of the datasets collected by their public health surveillance services. While these dataset could be invaluable for scientific research as well as epidemic intelligence and early-warning services, national legal frameworks and operational procedures limit sharing even between public health agencies. Increasingly challenged by open data initiatives in the public health domain, real-time data sharing could enable faster and better coordinated response during emergencies while opening new frontiers for data-driven interdisciplinary research in public health (23).

## Challenges of Data Sharing for Research

There are a number of challenges and potentially negative consequences to be addressed by new policies and regulations, through technical achievements and evidence-based healthcare interventions.

In addition to individual privacy discussed in detail in the next section, the high noise of large datasets is a major challenge requiring new analytics methods. Current methods still lack the level of robustness needed, resulting in misinterpretations and generation of false positive signals. Data security for large distributed infrastructures also requires rethinking our understanding of privacy and control and designing novel, secure computer system ecosystems. Data control is key to the success of computing approaches that underpin the digital economy (24).

Increased health interventions without clinically proven outcomes become a risk when research outcomes from large datasets are used to identify user-served individuals and disease areas for interventions ahead of the scientific evidence (for example, cervical screening prophylaxis occurring annually in the US).

Traditionally valued GP-individual relationships, based on the notion of family physicians was changed due to more frequent moves of citizens and new pressures on GPs to collect more “quality data” with technologies – enabling not only care improvement but also quality monitoring and GP remuneration. For multiple reasons, healthcare sectors in the UK and Europe are witnessing changes in delivery and continuity of care. Once a social contract between healthcare services and individuals it is now a relationship that takes profit into consideration.

## Integrating Data to Deliver Health-Care Benefits

Healthcare research on Big Data not only creates numerous opportunities but also brings new challenges – in particular, large storage, real-time analytics, and secure integration of distributed datasets.

Emerging data federation technologies enable new data sharing models across distributed data sources of information (internal or cloud-based sources). Data virtualization technologies (25) make it possible to run real-time analytics over high volume of distributed data while enforcing a robust security policy (data governance). Data do not need to be moved into a single location (as in the traditional approach) but remain in a repository while thousands of parallel queries can access them. This approach has been used successfully by pharmaceutical companies to accelerate their research projects and bring agility to the data scientists’ use of diverse data sources [such as at Pfizer (26)].

Encouraging examples are emerging from low- and middle-income settings. The INDEPTH network of autonomous population health and demographic surveillance sites throughout Africa and Asia launched its iShare initiative (www.indepth-ishare.org) in 2009, whereby several sites share almost 12.5 million person years of observational data from settings where little other population health data exist. Each of the participating sites is an independent organization. Yet through coordination by the southern-led INDPETH network, it has been possible to agree on processes, core minimum micro-dataset specifications, and conditions of use that overcome individual institutional constraints to make the most of available data to improve understandings of health and delivery of services. Leadership and finance are crucial to such initiatives and need to reflect local technical capacity and explicit strategies to recognize and respond to differing individual citizen, data-producer, and data-user needs.

In addition, harnessing the potential of real-time geo-located “unconventional” data sources, such as social media streams, loyalty cards, GPS-enabled mobile apps, and search queries adds another complex layer of privacy challenges. Never has so much data about so many people been held by so few with little policy and legal oversight and regulation, such as the case of IT companies and mobile data-driven start-ups (27). These data sources need to be made available for research, regardless of where the data comes from and by whom it has been collected, or its potential will never be realized for the individual user nor for the public good, while respecting valued private information about citizens, e.g., their current location (28).

## Balancing Access to Data with Individual Privacy

Individual’s attitudes towards healthcare services have undergone a major social and cultural shift over the last two decades, bringing new insights and attitudes to individuals’ privacy.

As discussed above, there are two sharply distinct approaches: traditional government healthcare and non-government research datasets, and novel user-generated personalized data held predominantly by industries.

Traditionally, an individual was a part of a healthcare system through an accepted social contract, implying rights as well as responsibilities for consent, protection, and privacy. In the new context of “consumer” healthcare services, research needs to renegotiate rights to use of data. This involves trust as well as technical security measures (29). Without this the potentially beneficial evidence for individuals and for populations as a whole would not be unlocked.

Attitudes against sharing medical information can stem from confusing messages and lack of controls in the past, as well as fear of the data being shared with third parties invading privacy and enabling personal data exploitation against individual’s interests, for example, with an insurance provider. Rather than an abstract notion of how data sharing may benefit person-kind, citizens’ engagement needs to be specific and honest about the risks and benefits of data sharing. While at the policy level, steps toward increasing transparency of data governance and de-identification techniques preserving meaningful usability of data for research are the key challenges (rather than anonymization with might make the data meaningless as a result). Transparency and open dialog with citizens are paramount for regaining public trust and setting cornerstones for a balanced agenda.

A striking contrast is provided by person physiological and medical data which is collected in vast quantities through social medial, wearable and tracking devices, MedTech and geo-located mobile apps (30). While there are differences in the legal environment in Europe and the US, without much awareness and concerns by users and in absence of policy debate, personal data are subject to industry-defined terms of conditions often with no opt-out clauses allowing use for personalized online/mobile marketing, internal research, and sale to third parties that could be in breach of the requirements of the Data Protection Act fair processing [as the recent example of OKCupid experiment and Uber provision of transport data to the Boston municipality (31)]. Much needed oversight, international government regulation and restoration of user control of personal data are essential to rebalance the current situation.

## Developing Responsibly Big Data and Open Data for Health

Public and business engagement in data regulation debates is essential for delivering better health outcomes. Risk-adverse regulatory authorities should be challenged by citizens and the research communities to engage in setting a balanced agenda that would benefit citizens and research communities.

- Public and citizen engagement: wider public awareness campaigns about the benefits and risks of sharing data could have positive effects but should be based on evidence and empirical methods while providing “success stories.” This will inevitably be a long-term process of developing an ongoing dialog with public, private sector and policymakers while increasing citizens trust in the government and understanding of data usage for research for public good. True media engagement reinforcing this dialog across the media spectrum rather than reversing the process through sensational coverage might be very challenging to establish.
- Clarity and transparency: data transparency and terms of use require a shared goal setting the core principles and establishing a regulation process that is fit for purpose. Transparency and clarity implies regulations and enforcement and also needs to be extended to public understanding of benefits and risks of data sharing (as defined by the Fundamental requirement for DPA Principle 1), strong disclosure, and notification mechanism informing public about potential violations. While there is little to disagree with the practical implementation of reinforcement of these principles is a different matter.
- New regulatory framework: a radical shift in the direction of regulation of data usage by industry should be developed. For Big Data, businesses giving control back to users generating the data (who could decide to sell the data back to the IT companies, for example) would require a very radical shift in existing business models – with the increasing closeness of big businesses to governments, perhaps, the biggest challenge among these to address at the moment.
- New data structures and Big Data analytics: common interoperability standards and new information sharing federal architectures for better Big Data storage and real-time analytics are required to deliver solutions that benefit individuals, practitioners, and healthcare professionals at every level. Transparently regulated third-party run data registries might provide an answer to the need for safe personal data repositories while offering access to data to authorized parties in machine readable formats (over an API, for example). Engagement of computer scientists and strong support for interdisciplinary collaborations should be championed.
- Training and education: citizens and healthcare professionals need to be better equipped with computing and ethical skills to enable future workforces to take full advantage of the digital revolution. Creating centers of excellence training future researchers and medical professionals in expertise in Big Data and open data – creating an essential interdisciplinary workforce should be a priority. Furthermore, training should also enable citizens and local communities in national and international settings to address local problems and draw from community needs.

## Conclusion and Key Recommendations

The potential of opening healthcare data and sharing big datasets is enormous – but the challenges and barriers to achieve this goal are enormous. As transparent access to Big Data is the key challenge for healthcare research on clinical and population research datasets, policymakers, and scientific and business communities should embrace the underlying challenges of a political and legal nature. Finding novel approaches to satisfy business interests and actively engage the public are essential for opening avenues to a balanced equilibrium: transparent data access for research needs and large-scale integrations preserving individual privacy. Technological advances on data sharing and transparency need to be driven by interdisciplinary research and translated into training of the future workforce.

Ultimately, healthcare policymakers at international level need to develop a shared policy and regulatory framework supporting a balanced agenda that safeguards personal information, limits business exploitations, and gives out a clear message to the public while enabling the use of data for research and commercial use. This would potentially improve the health of millions.

In lights of the risk-adverse pro-business policy making attitudes in this domain, it is the golden opportunity and professional responsibility of the research community to challenge policymakers and regulatory bodies authorities and actively lead on the complex multi-stakeholder processes of establishing this new agenda.

## Author Contributions

The high profile open data event involved 13 invited panelists and was cobadged with i-sense, the EPSRC IRC in Early Warning Sensing Systems for Infectious Diseases. Chaired by Sir John Tooke, the UCL Vice-Provost (Health), the 2 hours debate covered a broad range of themes representing views from the key stakeholders, including government, policymakers, NHS, academia, and industry. The initial proposal was to address the following five questions, but due to time constraints, only the first four were discussed. This paper was written up by Dr. Patty Kostkova with input provided by the panelists.

Q1. What are the benefits of opening up clinical data for health research?
Q2. How can data from different sources (public and private, including non-traditional sources) be merged to deliver health-care benefits?
Q3. How do we balance access to data with individual privacy?
Q4. What policy changes are needed to responsibly develop Big Data for health?
Q5. What are the lessons learned from care.data?

## Conflict of Interest Statement

The authors declare that the research was conducted in the absence of any commercial or financial relationships that could be construed as a potential conflict of interest.

## Footnotes

^1^http://quantifiedself.com/
